# Distinct PD-L1 binding characteristics of therapeutic monoclonal antibody durvalumab

**DOI:** 10.1007/s13238-017-0412-8

**Published:** 2017-05-09

**Authors:** Shuguang Tan, Kefang Liu, Yan Chai, Catherine W.-H. Zhang, Shan Gao, George F. Gao, Jianxun Qi

**Affiliations:** 1 CAS Key Laboratory of Pathogenic Microbiology and Immunology, Institute of Microbiology, Chinese Academy of Sciences, Beijing, 100101 China; 2 National Institute for Viral Disease Control and Prevention, Chinese Center for Disease Control and Prevention (China CDC), Beijing, 102206 China; 3 College of Laboratory Medicine and Life Sciences, Wenzhou Medical University, Wenzhou, 325035 China; 4 ImmuFuCell Biotechnology Co.Ltd., Beijing, 100102 China; 5 CAS Key Laboratory of Bio-medical Diagnostic, Suzhou Institute of Biomedical Engineering and Technology, Chinese Academy of Sciences, Suzhou, 215163 China

**Dear Editor**,

Blockade of PD-1/PD-L1 signaling pathway by monoclonal antibodies (MAbs) to release the anti-tumor activity of pre-existing tumor specific T cell immunity has initiated a new era for tumor immunotherapy. Administration of anti-PD-1 MAbs (nivolumab and pembrolizumab) in either monotherapy or in combination with anti-CTLA-4 MAbs or traditional chemotherapy has achieved a tumor regression rate of 30%–50% in dealing with melanoma, non-small cell lung cancer, etc. (Larkin et al., 2015). The approval of anti-PD-L1 atezolizumab and avelumab by US Food and Drug Administration (FDA) since 2016 has provided additional choices in dealing with multiple tumors aside from anti-PD-1 and anti-CTLA-4 MAbs as immunotherapeutic medication. The structures of the two therapeutic anti-PD-1 MAbs, nivolumab and pembrolizumab, complexed with PD-1 have been reported which elucidated the molecular basis of MAb-based anti-PD-1 immunotherapy (Tan et al., 2016a, b; Na et al., 2017; Tan et al., 2017). Complex structures of avelumab and BMS-936559 with PD-L1 were also reported which contributes a better understanding of the molecular basis of MAb-based anti-PD-L1 checkpoint blockade therapy (Lee et al., 2016; Liu et al., 2017). In addition, two additional anti-PD-L1 MAbs are in clinics or phase III trials, atezolizumab and durvalumab. Durvalumab (MEDI4736) is a fully human IgG1 MAb targeting PD-L1 that was developed by AstraZeneca, and has been approved by US FDA very recently. Multiple Phase III clinical trials are still ongoing in non-small cell lung cancer, head and neck cancer, urothelial cancer, etc. (NCT02542293, NCT02369874, NCT02516241, etc.). A Phase Ib report demonstrated that durvalumab is well tolerated and showed promising anti-tumor efficacy in non-small cell lung cancer patients (Antonia et al., 2016). However, the molecular basis of durvalumab-based anti-PD-L1 reactivity and binding characteristics compared to the other three MAbs used in clinics has not yet been elucidated.

In the present study, we expressed the two-Ig-domain PD-L1 and single chain Fv fragment (scFv) of durvalumab as inclusion bodies in *E* *scherichia* *coli* cells. Soluble proteins were obtained by *in vitro* refolding, and the two refolded proteins survived well in gel filtration (Fig. S1). Subsequently, crystal screen was performed with the durvalumab-scFv/PD-L1 complex proteins, and well-diffractable crystals grew in 3.5 mol/L sodium formate, pH 7.0 (See more details in supplementary information).

The complex structure of durvalumab-scFv/PD-L1 was determined by molecular replacement at a resolution of 2.3 Å (Table S1). The binding of durvalumab to PD-L1 involves both of its heavy chain (VH) and light chain (VL) (Fig. 1A). All of the three complementarity-determining regions (CDRs) of VH and CDR1 and CDR3 of VL contribute to interactions with PD-L1, leaving LCDR2 without any contacts. Previous reports on the anti-PD-1 MAbs revealed that the binding of these MAb is mainly located on the loops of PD-1, i.e., the N-terminal loop of PD-1 for nivolumab interaction and the C’D loop for pembrolizumab. However, the binding of avelumab and BMS-936559 is mainly located on the strands of the front-β-sheet face of PD-L1. Here, the binding of durvalumab on PD-L1 was also mainly located on the front β-sheet face which is constituted by A, G, F, C, and C’ strands of the IgV domain of PD-L1. A detailed analysis of the interactions between durvalumab and PD-L1 shows an unbiased contribution from VH and VL of durvalumab in binding to PD-L1. The A, G, and F strands of PD-L1 provide major hydrogen bond interactions with durvalumab (Fig. 1B). D26 of the A strand and R113 of the F strand of PD-L1 were occupied by S30 of LCDR1 and E58 of HCDR2, respectively (Table S2). Especially, residues of the G strand (Y123, K124, and R125) provide multiple hydrogen bonds to both VH (F104 of HCDR3 and N51 nearby HCDR2) and VL (Y92 and S94 of LCDR3), which contribute major hydrogen bond interactions to durvalumab, 7 out of 10 hydrogen bonds in all (Table S2).

**Figure 1:**
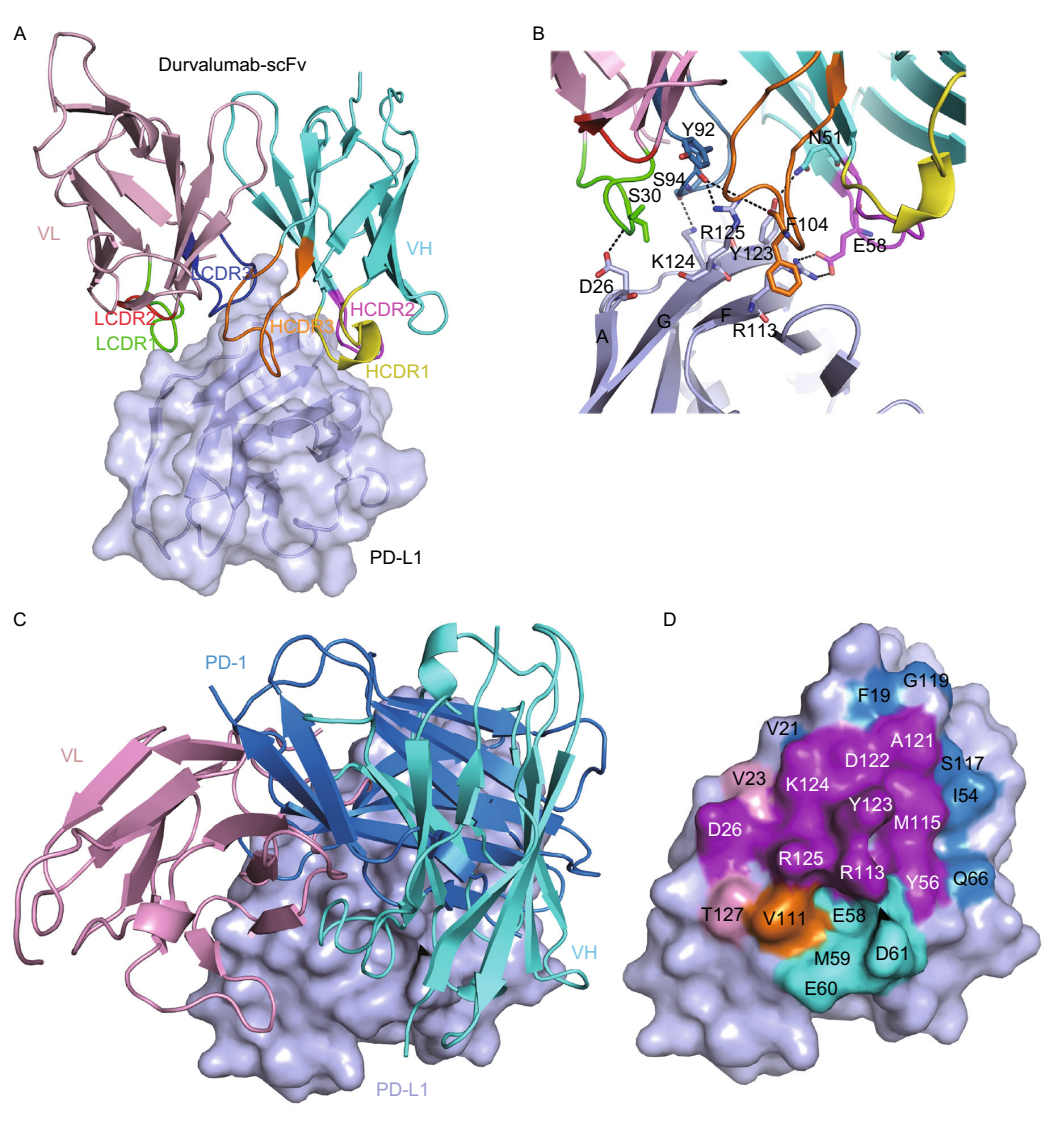
**Structural basis of durvalumab-based binding to PD-L1 and intervention of the PD-1/PD-L1 interaction**. (A) Overall structure of durvalumab-scFv/PD-L1 complex. The MAb-binding IgV domain of PD-L1 is shown as surface diagram in light blue, and the heavy (VH) and light chains (VL) of scFv are shown as cartoon representations in cyan and light pink, respectively. The CDR1, CDR2, and CDR3 loops of VH are colored in yellow, magenta and orange, respectively. The CDR1, CDR2, and CDR3 loops of VL are colored in green, hot pink and blue, respectively. (B) Detailed interactions in the durvalumab-scFv/PD-L1 interface. Residues involved in the hydrogen bond interaction are shown as sticks and labeled. Hydrogen bonds are shown as dashed lines. (C) Superimposition of the durvalumab-scFv/PD-L1 complex structure with the PD-1/PD-L1 complex structure (PDB: 4ZQK). PD-1 is shown in marine, PD-L1 as a surface diagram in light blue, and durvalumab-scFv VL in light pink, VH in cyan, respectively. (D) Binding surface on PD-L1 for PD-1 or durvalumab. The binding residues of PD-1 on PD-L1 are colored in marine, whereas residues contacted by the durvalumab-scFv VH or VL are colored in cyan or light pink, respectively, and the overlapping residues bound by both the receptor PD-1 and durvalumab are colored in purple

Structural superimposition of the PD-1/PD-L1 complex (PDB: 4ZQK) and the durvalumab-scFv/PD-L1 complex was conducted to elucidate the molecular basis of durvalumab-based PD-1/PD-L1 intervention. The binding of durvalumab to PD-L1 shows a stereo clash with that of PD-1 (Fig. 1C). The binding surface of durvalumab and PD-1 on PD-L1 is highly overlapped that residues of PD-L1, which contributed major hydrogen bond interactions with PD-1 (D26, Y123, K124, and R125), were also occupied by durvalumab (Fig. 1D) (Zak et al., 2015). The competitive binding involves both VH and VL of durvalumab. Thus, the molecular basis of durvalumab-based PD-1/PD-L1 blockade is that the unbiased binding of durvalumab VH and VL to PD-L1 provides steric clash to abrogate the binding of PD-1/PD-L1. This is quite different from anti-PD-1 MAbs that the residues of PD-1 contributing to major contacts with anti-PD-1 MAbs, in both nivolumab and pembrolizumab, were different from the residues participating in competitive binding to the ligand (Na et al., 2017; Tan et al., 2017).

To date, there are three additional anti-PD-L1 MAbs used in clinics in addition to durvalumab, i.e., avelumab, atezolizumab, and BMS-936559. The determination of the complex structures of avelumab and BMS-936559 with PD-L1 has enabled us to compare the binding characteristics of these MAbs to elucidate the rules of interaction between therapeutic MAbs and PD-L1 (Lee et al., 2016; Liu et al., 2017). Overall, these three MAbs bind to PD-L1 from distinct orientations which show stereo clash with that of PD-1, while the blockade contributions from the VH and VL of these MAbs are quite different (Fig. 2A). The blockade of avelumab and BMS-936559 to the interaction of PD-1/PD-L1 is mainly contributed by their VH domain while both VH and VL of durvalumab are involved in blockade of PD-1/PD-L1 interaction. The epitopes of these three MAbs on PD-L1 are quite different from each other but all show overlapped binding areas with that of PD-1 (Fig. 2B). These results indicate that though avelumab, BMS-936559, and durvalumab bind to PD-L1 with distinct binding interfaces, the binding of these MAbs would provide stereo clash to that of PD-1, and thus abrogate the binding of PD-1 to PD-L1. Moreover, the binding of any of these three MAbs to PD-L1 has stereo clash to the other MAbs, which suggests that co-administration of these three MAbs would not achieve additive efficacy. Furthermore, the binding kinetics of the four therapeutic anti-PD-L1 MAbs was investigated using surface plasmon resonance (SPR) analysis in this study. PD-L1 protein was immobilized on the chip, while scFvs of the four anti-PD-L1 MAbs were flowed through. The results reveal that durvalumab, atezolizumab, and BMS-936559 show similar binding affinity to PD-L1, with *K*_D_ = 0.667 nmol/L, 1.75 nmol/L, and 0.83 nmol/L, respectively (Fig. 2C and Table S3). However, BMS-936559 shows a much higher association rate constant (*K*_a_, 1.05 × 10^6^/ms) and/or dissociation rate constant (*K*_d_, 8.68 × 10^−4^/s) compared to that of durvalumab (*K*_a_ = 4.28 × 10^5^/ms, *K*_d_ = 2.85 × 10^−4^/s) and atezolizumab (*K*_a_ = 8.93 × 10^4^/ms, *K*_d_ = 1.56 × 10^−4^/s). On the other hand, the binding affinity of avelumab to PD-L1 is much higher than the other three MAbs (*K*_D_ = 0.0467 nmol/L) with highest *K*_a_ and lowest *K*_d_ value (Table S3). Taken together, the binding kinetics of durvalumab is much similar to that of atezolizumab, while binding properties of BMS-936559 and avelumab are distinct from the above two MAbs. The differential binding profiles of these anti-PD-L1 MAbs would have profound effects on their pharmacokinetics. However, whether the distinct binding epitopes and kinetics of these MAbs would affect therapeutic efficacy remains to be elucidated. Escape from anti-PD-1 or anti-PD-L1 could be induced from multiple aspects, including selection of poor-immunogenic tumor cells, immune evasion via up-regulation of alternative inhibitory molecules, etc. (Kim and Chen, 2016). Though the mechanisms of escape from anti-PD-1/PD-L1 remain mostly unknown, one possible concern is that the administration of anti-PD-L1 MAbs would increase the possibility of MAb driven off-target escape derived from PD-L1 mutation or alternative splicing driven isotype switch. The distinct binding kinetics of the four anti-PD-L1 MAbs may differ in MAb-driven off-target escape, and thereafter duration of anti-tumor efficacy and reduction of relapse. Another issue that should be taken into consideration is their IgG subclasses. Durvalumab, atezolizumab, and avelumab are IgG1 subclass while BMS-936559 is IgG4, which indicates the differences in Fc-mediated antibody-dependent cell-mediated cytotoxicity (ADCC) and complement dependent cytotoxicity (CDC). Therefore, further investigations should be conducted to elucidate the molecular mechanisms of the differences in anti-tumor efficacy and duration of these four anti-PD-L1 MAbs.

**Figure 2:**
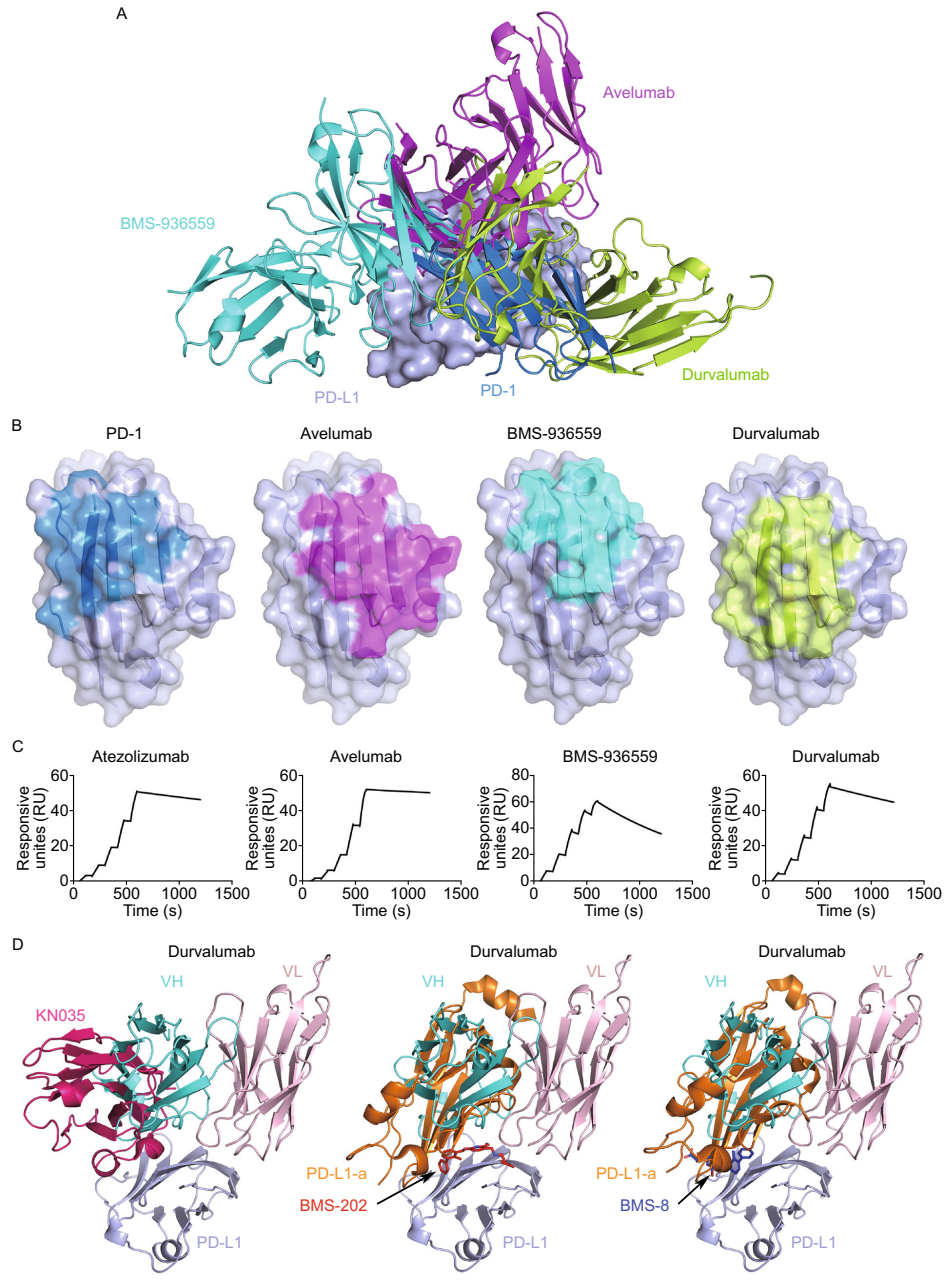
**Comparison of the binding mode and kinetics of anti-PD-L1 MAbs**. (A) Superimposition of PD-1/PD-L1 complex and PD-L1 complexed with avelumab (PDB: 5GRJ), BMS-936559 (PDB: 5GGT), and durvalumab. PD-1 is shown in marine, PD-L1 as a surface diagram in light blue, durvalumab in limon, avelumab in magenta and BMS-936559 in cyan, respectively. (B) Binding surface of PD-1 and binding epitopes of avelumab, BMS-936559, and durvalumab on PD-L1. (C) SPR analysis of the binding kinetics of anti-PD-L1 MAbs and PD-L1 using a BIAcoreT100 system. The data presented here are representative of three independent experiments with similar results. (D) Superimposition of the anti-PD-L1 nanobody (KN035)/PD-L1 complex and complex structures of homodimeric PD-L1 and small-molecule inhibitors, BMS-202 and BMS-8, with durvalumab-scFv/PD-L1. KN035 is shown in hot pink while VL and VH of durvalumab-scFv are presented in light pink and cyan, respectively. The second PD-L1 (PD-L1-a) is presented in orange while small-molecule inhibitors BMS-202 and BMS-8 are shown as sticks in red and blue, respectively

The efficacy of MAb-based PD-1/PD-L1 blockade treatment strongly depends on the accession of MAbs to PD-1 or PD-L1 in the tumor microenvironment (Maute et al., 2015; Palucka and Coussens, 2016). The less efficient penetration of MAbs because of their large size and the complexity of the tumor microenvironment has impelled the development of small-molecule biologics with superior penetration efficiencies. Complex structures of PD-L1 with a PD-L1 targeting nanobody and non-peptide small inhibitors have been reported (Zak et al., 2016; Zhang et al., 2017). This enabled us to compare the binding properties of these small-molecule inhibitors with durvalumab to investigate the hot spots on PD-L1 for future development of biologics for a better anti-tumor efficacy. The results revealed that the binding of anti-PD-L1 nanobody (KN035) shows more similarity to that of VH domain of durvalumab (Fig. 2D). Similarly, the second PD-L1 molecule induced by the binding of non-pepitde anti-PD-L1 inhibitors, the BMS-202 and BMS-8, which is believed to be responsible for the blockade of PD-1/PD-L1 interaction, resembles the binding of VH domain of durvalumab (Fig. 2D). On the other hand, the binding of VL does not overlap with that of the nanobody and small-molecule inhibitors. These results indicate that the binding sites of the VH domain of durvalumab may serve as hot spots for small inhibitory biologics development.

In summary, we report the structural basis of durvalumab-based binding to PD-L1 and the molecular mechanisms of PD-1/PD-L1 blockade. Moreover, a comparative study of the binding characteristics of anti-PD-L1 MAbs currently available in clinics and small-molecule inhibitors was conducted to elucidate the rules of binding properties of anti-PD-L1 biologics. The distinct binding interface and kinetics of durvalumab compared to the other therapeutic MAbs indicate the special anti-tumor efficacy and pharmacokinetics of this MAb. The remarkable similarity of the binding properties of VH domain of durvalumab with that of the anti-PD-L1 nanobody and non-peptide inhibitors suggests that the binding sites of VH may serve as hot spot for future anti-PD-L1 small-molecule inhibitor development.

## DATA DEPOSITION

Atomic coordinates have been deposited in the Protein Data Bank (PDB http://www.rcsb.org/pdb) under accession code 5XJ4.

## FOOTNOTES

This work was supported by the National Natural Science Foundation of China (Grant Nos. 31390432 and 31500722) and the National Basic Research Program (973 Program) (NO. 2013CB531502). We also thank Yuanyuan Chen and Zhenwei Yang from Institute of Biophysics, Chinese Academy of Sciences for their technical support in the SPR assay. G.F.G. is a leading principle investigator of NSFC Innovative Research Group (Grant No. 81621091).

## Electronic supplementary material

Below is the link to the electronic supplementary material.

Supplementary material 1 (PDF 371 kb)
