# Supplementary material for: Distinct PD-L1 binding characteristics of therapeutic monoclonal antibody durvalumab
Source: Protein Cell. 2017 May 9;9(1):135–9. doi: 10.1007/s13238-017-0412-8 (PMC5777972; doi:10.1007/s13238-017-0412-8)
Supplement: Supplementary file 1 — Supplementary material 1 (PDF 371 kb) [file 13238_2017_412_MOESM1_ESM.pdf]

Supplementary data

Figure S1

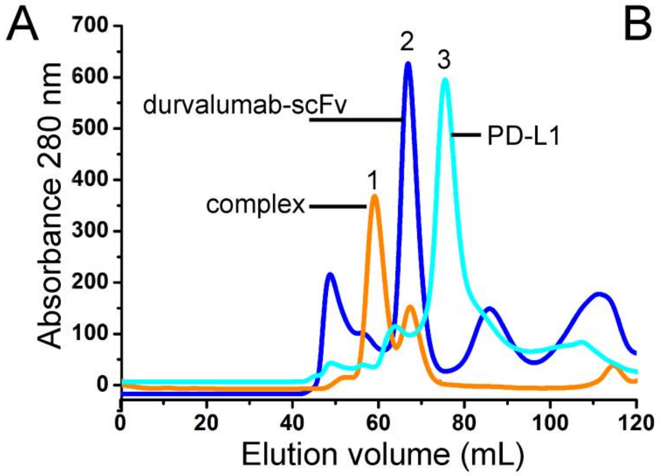

Figure S1. Survival of durvalumab-scFv and PD-L1.

A. Gel filtration profiles of PD-L1 (cyan)B, durvalumab-scFv (blue) and the durvalumab-scFv/PD-L1 complex (orange) were analysed by size-exclusion chromatography as indicated. The x axis represents the elution volume while Y axis shows the absorbance at 280 nm. **B.** SDS-PAGE confirms the formation of durvalumab-scFv/PD-L1 complex. Representative samples from gel filtration were loaded as indicated. The presence of durvalumab-scFv and PD-L1 in lane 2 confirms the complex protein after survival.

Table S1. Data collection and refinement statistics

Space group	P23
Wavelength (Å)	0.9791
Unit cell dimensions	

a, b, c (Å)	133.89, 133.89, 133.89
α, β, γ (°)	90.00, 90.00, 90.00
Resolution (Å)	50.00–2.30 (2.38–2.30)
Observed reflections	36086
Completeness (%)	100.00 (100.0)
Redundancy	21.3 (17.8)
R_{merge} (%)	11.2 (441.5)
I/σ	32.00 (1.53)
Refinement	
R_{work} / R_{free}(%)	21.58/24.03
No. atoms	
Protein	3457
Water	169
B-factors	
Protein	42.5
Water	37.2
r.m.s. deviation	
Bond lengths (Å)	0.004
Bond angles (°)	0.946
Ramachandran plot	
Favored (%)	94.2
Allowed (%)	5.8

Outliers (%)	0.0

[1] Values in parentheses are given for the highest resolution shell.

Table S2. Residues contributed interaction between durvalumab and PD-L1

	Antibody	PD-L1	Contacts	Total
VH	W34	R113, Y123	7, 8	189
	N51	Y123 (1)	3	
	K53	E58, R113	4, 3	
	E58	Y56, E58, R113 (2), M115, Y123	2, 1, 9, 9, 1	
	Y60	M115, A121, D122, Y123	2, 5, 2, 12	
	D63	M18	2	
	E100	Y123, R125	1	
	W103	E60, D61	35, 13	
	F104	E58, E60, M59, V111, Y112, R113, R125 (1)	8, 8, 5, 6, 3, 18, 11	
	G105	R125	6	
	E106	V111, R125	1, 3	
VL	S30.	V23, D26 (1)	1, 6	72
	S31	D26, R125, T127	12, 1, 1	
	Y33.	V111, R125, T127	1, 11, 1	

Y92	R125 (2)	6
G93	K124, R125	4, 2
S94	K124 (1)	6
L95.	D122, Y123	3, 5
W97	Y123, R125	6, 6

[1] Numbers represent the number of atom-to-atom contacts between the antibody residues and the PD-L1 residues, which were analyzed by the Contact program in CCP4 suite (the distance cutoff is 4.5Å).

[2] Numbers in the parentheses represent the number of hydrogen bonds between the antibody residues and the PD-L1 residues, which were analyzed by the Contact program in CCP4 suite (the distance cutoff is 3.5 Å).

Table S3. Binding profiles of the four MAbs to PD-L1

MAbs	ka[1] (10^4/Ms)	kd[2](10^{-4}/s)	KD (nM)
atezolizumab	8.93	1.56	1.75
durvalumab	42.8	2.85	0.667
avelumab	161	0.753	0.0467
BMS-936559	105	8.68	0.83

[1] ka, association rate constant.

[2] kd, dissociation rate constant.

Materials and methods

Refolding and purification of PD-L1 and scFv of the four anti-PD-L1 MAbs

PD-L1 extracellular domain fragment (from F19 to R238) was over-expressed as inclusion bodies in the BL21 (DE3) strain of *E. coli*. Renaturation and purification of PD-L1 was performed as previously described (Liu et al., 2017). Briefly, inclusion bodies of PD-L1 was diluted against a refolding buffer (100 mM Tris, pH 8.0; 400 mM L-Arginine; 5 mM EDTA-Na; 5 mM Glutathione (GSH); 0.5 mM Glutathione disulfide (GSSG)) at 4°C for 24 h. Then the PD-L1 was concentrated and exchanged into a buffer of 20 mM Tris-HCl (pH 8.0) and 15 mM NaCl and further analyzed by HiLoad 16/60 Superdex 75 pg (GE Healthcare) chromatography. Durvalumab-scFv was constructed as VL-$(GGGGS)_4$-VH as previously described and cloned into the pET21a expression vector (Invitrogen) (Liu et al., 2017). The durvalumab-scFv was over expressed in *E. coli* as inclusion bodies. Renaturation and purification of durvalumab-scFv was performed as the PD-L1 protein. The PD-L1 and durvalumab-scFv were then mixed together at a molar ratio of 1:2 and incubated for 1 h on ice. The PD-L1 and avelumab-scFv complex was further purified by HiLoad 16/60 Superdex 75 pg (GE Healthcare) chromatography. The proteins of avalumab–scFv, atezolizumab-scfv and BMS-936559-scFv were all obtained as durvalumab-scFv.

Crystal screening of the durvalumab-scFv/PD-L1 complex

The purified durvalumab-scFv/PD-L1 complex protein was adjusted to a concentration of 15 mg/mL in 20 mM Tris-HCl (pH 8.0) and 15 mM NaCl buffer.

Crystals were screened by the vapour-diffusion sitting-drop method. Normally, 0.3 μL complex protein and 0.3 μL reservoir solution were mixed, sealed and equilibrated against 100 μL reservoir solution at 4 ℃. Crystals of durvalumab-scFv/PD–L1 complex grew in 3.5 M sodium formate (pH 7.0). For cryoprotection, crystals were transferred to reservoir solutions containing 20% glycerol.

Data collection and structure determination

The complex structure was solved by molecular replacement method (Otwinowski and Minor, 1997) using Phaser from the CCP4 program suite, with the reported PD-L1 structure (PDB: 3RRQ) and Fab tructure (PDB: 3EYQ) as the search models (Bailey, 1994). COOT and PHENIX were used or subsequent model building and refinement (Emsley and Cowtan, 2004; Adams et al., 2010). The stereochemical qualities of the final model were assessed with MolProbity (Chen et al., 2010). Data collection and refinement statistics are summarized in Table S1. All structure figures were prepared with pymol (http://www.pymol.org).

SPR experiments

The affinity and binding kinetics of PD-L1 with durvalumab-scFv, avalumab–scFv, atezolizumab-scfv or BMS-936559-scFv were analyzed at 25 ℃ by using BIAcoreT100 system with CM5 chips (GE Healthcare). HBS-EP buffer consisting of 150 mM NaCl, 10 mM HEPES, pH 7.4 and 0.005% (v/v) Tween-20 was used as the kinetics analysis buffer and all proteins were exchanged into this buffer in advance through gel filtration. Approximately 1000 responsive units of PD–L1 were immobilized on the chip, and a blank channel was used as negative control. Gradient

concentrations of the MAb-scFv ranging from 5 nM to 80 nM were then flowed through the chip surface, and the responsive units were measured. After each cycle, the sensor surface was regenerated with 10 mM glycine (pH1.7). The binding kinetics were all analyzed with the software of BIA evaluation Version 4.1 using a 1:1 Langmuir-binding model.

References

Adams, P.D., Afonine, P.V., Bunkoczi, G., Chen, V.B., Davis, I.W., Echols, N., Headd, J.J., Hung, L.W., Kapral, G.J., Grosse-Kunstleve, R.W., *et al.* (2010). PHENIX: a comprehensive Python-based system for macromolecular structure solution. Acta Crystallogr D Biol Crystallogr 66, 213-221.

Bailey, S. (1994). The Ccp4 Suite - Programs for Protein Crystallography. Acta Crystallogr D Biol Crystallogr 50, 760-763.

Chen, V.B., Arendall, W.B., 3rd, Headd, J.J., Keedy, D.A., Immormino, R.M., Kapral, G.J., Murray, L.W., Richardson, J.S., and Richardson, D.C. (2010). MolProbity: all-atom structure validation for macromolecular crystallography. Acta Crystallogr D Biol Crystallogr 66, 12-21.

Emsley, P., and Cowtan, K. (2004). Coot: model-building tools for molecular graphics. Acta Crystallogr D Biol Crystallogr 60, 2126-2132.

Liu, K., Tan, S., Chai, Y., Chen, D., Song, H., Zhang, C.W., Shi, Y., Liu, J., Tan, W., Lyu, J., *et al.* (2017). Structural basis of anti-PD-L1 monoclonal antibody avelumab for tumor therapy. Cell Res 27, 151-153.

Otwinowski, Z., and Minor, W. (1997). Processing of X-ray diffraction data collected in oscillation mode. Methods Enzymol 276, 307-326.
